# Exploring biomarkers and regulatory mechanisms associated with 20 protein post-translational modifications in acute myocardial infarction based on transcriptomics

**DOI:** 10.3389/fcvm.2026.1848328

**Published:** 2026-10-21

**Authors:** Jie Zhang, Binhe Li, Mengyue Yang, Jingchao Lu, Huiqing Jiang, Fei Chen

**Affiliations:** 1 Department of Cardiology, The Second Hospital of Hebei Medical University, Shijiazhuang, Hebei, China; 2 Department of Gastroenterology, The Second Hospital of Hebei Medical University, Shijiazhuang, Hebei, China

**Keywords:** acute myocardial infarction, biomarkers, immunity, protein post-translational modification, transcriptomics

## Abstract

**Background:**

Acute myocardial infarction (AMI) is a critical condition resulting from coronary occlusion, leading to myocardial necrosis and dysfunction. Protein posttranslational modifications (PTMs) regulate key inflammatory and signaling pathways in its pathogenesis. Accordingly, exploring transcriptomic signatures of genes encoding PTM-regulatory factors may provide valuable insights for improving early diagnosis and therapeutic stratification in AMI.

**Methods:**

Candidate genes were obtained by integrating transcriptomic data on AMI from public databases with a previously published gene set encoding regulators for 20 categories of protein PTMs. Differentially expressed genes (DEGs) were intersected with this PTM-regulator gene set to identify PTM-related DEGs. Machine learning approaches together with expression analyses were employed to identify diagnostic biomarkers. Their potential mechanisms were systematically elucidated via multidimensional analyses, including deep neural network (DNN) model construction, biological pathway analysis, immune landscape profiling, regulatory network reconstruction, and therapeutic compound prioritization. Clinical validation of the key biomarkers was performed using RT–qPCR.

**Results:**

This study identified three biomarkers among PTM-regulator-related genes for AMI: *GABARAPL1*, *PELI1*, and *NLRP3*. Enrichment profiling showed that all three biomarkers were prominently linked to pathways related to Leishmania infection. Correlation analysis of immune cell profiles showed that all three biomarkers were most strongly associated with plasmacytoid dendritic cells. Molecular regulatory network analysis uncovered their associated transcription factors, targeted miRNAs, and lncRNA interaction networks. Drug prediction and molecular docking identified several potential targeting compounds, among which 1,4-chrysenequinone exhibited docking scores of −7 kcal/mol and −8.9 kcal/mol with *GABARAPL1* and *PELI1*, respectively, while calcitriol showed a docking score of −10.1 kcal/mol with NLRP3, indicating strong predicted binding affinity. RT-qPCR clinical validation confirmed significant upregulation of *GABARAPL1*, *PELI1*, and *NLRP3* in AMI patients (all *P* < 0.01), consistent with the integrative multi-omics findings.

**Conclusion:**

Using transcriptome-based bioinformatics, this study identified and validated three transcriptomic biomarkers (*GABARAPL1*, *PELI1*, and *NLRP3*) among genes encoding PTM-regulatory factors for AMI, offering molecular resources for future investigations into diagnostic and therapeutic targets in AMI.

## 1 Introduction

Acute myocardial infarction (AMI) is a condition characterized by a sudden interruption of blood flow in the coronary arteries, leading to severe and sustained ischemia and subsequent necrosis of the myocardial tissue in the affected region (1). As a leading cause of mortality worldwide, AMI is more prevalent in older adults and men and is increasingly affecting younger individuals, thereby imposing a substantial public health burden (2). The hallmark of AMI is irreversible damage to myocardial cells, accompanied by characteristic electrocardiogram (ECG) changes and significant elevations in myocardial injury biomarkers (3). The primary etiology is rupture of atherosclerotic plaques in the coronary arteries, leading to acute vessel occlusion due to thrombosis (4). The typical clinical presentation includes sudden, severe, and persistent chest pain often radiating to the left shoulder or jaw, accompanied by diaphoresis, nausea, and dyspnea. However, some older adults or patients with diabetes may present with atypical symptoms, which complicate the diagnostic process (5). The current therapeutic priority lies in the rapid achievement of myocardial reperfusion, primarily through percutaneous coronary intervention or thrombolytic therapy. However, risks such as reperfusion injury, heart failure, and arrhythmias remain significant clinical challenges (6). Cardiac troponin T (cTnT) is currently recognized as the benchmark biomarker for the clinical diagnosis of AMI. However, its clinical utility remains subject to certain confounding factors. Chest compressions during cardiopulmonary resuscitation (CPR) may induce myocardial injury, leading to elevated cTnT release and thereby confounding the diagnostic interpretation of AMI (7). Furthermore, the timing of blood sampling and the choice of sampling sites have been shown to significantly affect the analytical accuracy of cTnT measurements (8). Collectively, these factors limit the diagnostic reliability of cTnT in specific clinical scenarios. Therefore, identifying highly sensitive and specific biomarkers is crucial for the early recognition of AMI.

Posttranslational modifications (PTMs) are extensively involved in key pathological processes in cardiovascular diseases, including cellular stress, inflammatory signaling, and damage repair, by modulating protein activity, stability, and interactions (9). This involvement provides a valuable perspective and theoretical foundation for elucidating the molecular pathology of AMI and identifying novel biomarkers with mechanistic support. PTMs encompass diverse covalent alterations that are added to proteins following their synthesis. Common types of PTMs include phosphorylation, acetylation, and ubiquitination (10, 11). These modifications can significantly influence protein structure, activity, stability, subcellular localization, and interactions, thereby regulating virtually all cellular processes and intercellular communication (12). In the field of cardiovascular diseases, the regulatory role of PTMs in myocardial injury and repair has attracted increasing attention. Accumulating evidence indicates that distinct PTM events are closely involved in the development and progression of AMI and its associated complications. For example, protein SUMOylation, as an important PTM, has been demonstrated to exert cardioprotective effects. Specifically, the protein inhibitor of activated STAT1 (PIAS1) can enhance PPAR*γ* SUMOylation while inhibiting NF–*κ*B activity, thereby exerting antiapoptotic and anti-inflammatory effects and alleviating myocardial ischemia–reperfusion injury (MIRI), a key determinant of AMI prognosis (13). This mechanism reveals a potential therapeutic pathway. In addition, 34 guanine sites associated with AMI have been identified in previous studies, suggesting potential synergy between inflammatory pathways and PTMs (14). However, many roles of PTMs in AMI remain poorly understood, which not only limits our understanding of the underlying pathophysiological mechanisms of AMI but also hinders the development of PTM-based diagnostic biomarkers and targeted therapeutic strategies.

Despite accumulating evidence implicating PTMs in cardiovascular diseases, the specific roles and regulatory mechanisms of diverse PTM types in AMI remain largely unexplored. We hypothesized that integrating transcriptomic profiling with a published gene set of PTM-regulatory factors could help identify putative transcriptomic biomarkers participating in PTM-governed pathological cascades in AMI. Of note, mRNA expression changes in these regulators cannot be equated to actual alterations in protein PTM events. Accordingly, this study aimed to systematically screen PTM-related biomarkers in AMI and explore their functional roles, immune associations, regulatory networks, and therapeutic potential. The detailed analysis workflow is illustrated in Figure 1.

**Figure 1:**
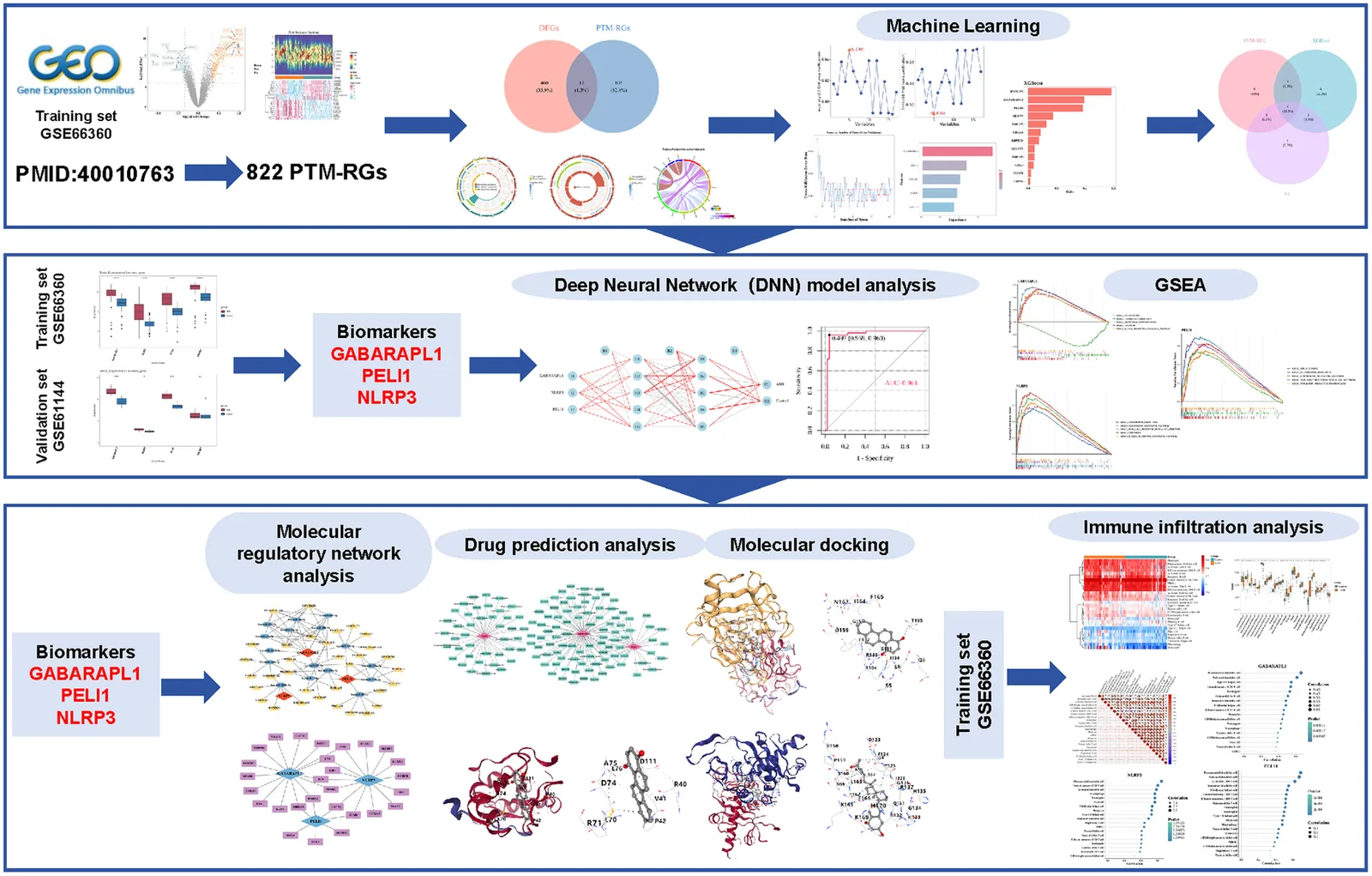
Analysis flowchart.

## 2 Materials and methods

### 2.1 Data source

Publicly available transcriptomic profiles were retrieved from the Gene Expression Omnibus (GEO) repository (https://www.ncbi.nlm.nih.gov/geo/). The dataset GSE66360 (platform: GPL570, accessed 16 June 2025), derived from microarray hybridization, comprises peripheral blood samples from 49 patients with AMI (AMI group) and 50 healthy controls (control group), and was used as the training set. In addition, the dataset GSE61144 (platform: GPL6106, accessed 16 June 2025), also derived from microarray hybridization, includes peripheral blood samples 7 patients with AMI (AMI group) and 10 healthy controls (control group) and was designated as the validation set. Furthermore, a previous study yielded 822 genes encoding regulators of 20 types of posttranslational modifications (PTM–RGs), which were utilized for subsequent biomarker identification and analysis (15).

### 2.2 Differential expression analysis

Differential expression profiling between AMI and control samples in the GSE66360 training cohort was performed using the R package “limma” (v 3.54.0) (16). Genes with an adjusted *P*-value <0.05 and an absolute log2 fold change (|log_2_FC|) greater than 1 were considered significantly differentially expressed. The top differentially regulated genes were visualized using a heatmap generated by the “ComplexHeatmap” (v 2.14.0) (17), highlighting the 10 most upregulated and 10 most downregulated transcripts ranked by |log_2_FC|. In parallel, a volcano plot was constructed to display the global distribution of DEGs and to annotate the most strongly altered genes.

### 2.3 Identification of candidate genes and their functional and interaction profiles

To further identify genes associated with the 20 types of protein PTMs in AMI, the intersection of DEGs and PTM–RGs was obtained using the R package “ggvenn” (v 1.7.3) (18). Subsequently, the biological roles of the candidate genes were explored by performing Gene Ontology (GO) and Kyoto Encyclopedia of Genes and Genomes (KEGG) pathway enrichment analyses using the R package “clusterProfiler” (v 4.2.2) (*P* < 0.05) (19), with the org.Hs.eg.db annotation database (version 3.16.0) serving as the reference background (20). To investigate protein–protein interactions among the candidate genes, a protein–protein interaction (PPI) network was constructed using the Search Tool for the Retrieval of Interacting Genes/Proteins (STRING) database (https://string-db.org/). A minimum required interaction confidence score of 0.15 was applied to filter protein–protein interactions.

### 2.4 Biomarker identification

To further screen biomarkers highly correlated with AMI from the candidate genes, three complementary machine learning strategies—support vector machine recursive feature elimination (SVM–RFE), extreme gradient boosting (XGBoost), and random forest (RF)—were implemented. Initially, the R package “e1071” (v 4.7-1.1) (21) was utilized to rank the genes obtained from the SVM algorithm iteratively, applying the RFE method (which reduces combinations of input genes stepwise to arrive at a single gene combination while computing importance rankings at each iteration), to identify the optimal gene set with the lowest error rate and highest accuracy as Feature Gene Set 1. Subsequently, based on the candidate genes, model training was conducted using the R package “XGBoost” (v 1.7.3.1) (22), setting the maximum tree depth to 2, learning rate to 0.3, objective function to binary logistic regression, and iterations to 20. After training, the feature importance genes were extracted using the xgb.importance function to obtain Feature Gene Set 2. Subsequently, RF analysis was conducted using the R package “randomForest” (v 4.7-1.1) (23), in which variable importance was evaluated by the mean decrease in the Gini index, reflecting the contribution of each gene to classification performance. The top 5 genes ranked by importance were designated as Feature Gene Set 3. Finally, the intersections of the feature gene sets obtained from the three algorithms were computed using the R package “ggvenn” (v 1.7.3) to identify candidate biomarkers. To validate their expression patterns, Wilcoxon rank-sum tests (*P* < 0.05) were applied to compare biomarker expression levels between AMI and control samples in both the GSE66360 training cohort and the GSE61144 validation cohort. Genes showing consistent and significant expression differences across both datasets were designated as the final biomarkers. Finally, to assess the diagnostic performance of biomarkers, the R package “pROC” (v 1.18.0) (24) was utilized to perform receiver operating characteristic (ROC) analysis.

### 2.5 Deep neural network model construction and validation

A deep neural network (DNN) model was developed to quantify the ability of the identified biomarkers to discriminate AMI samples from healthy controls, using all samples in the GSE66360 training cohort and the R package “neuralnet” (v 1.44.2) (25). The model input included the biomarkers, while optimization of the hidden layer structure and weight parameters was performed to establish classification decision boundaries for sample status discrimination. Model performance was evaluated by constructing an ROC curve using the R package “pROC” (v 3.4.1) (19), and the area under the ROC curve (AUC) was calculated to measure predictive accuracy. To avoid performance overestimation from training and evaluating the model using all samples from the raw GSE66360 dataset, fivefold stratified cross-validation was performed. Stratified random splitting was implemented by AMI and control groups to maintain balanced sample sizes of the two groups within each fold. In each iteration, four folds served as the training set, and the remaining fold was used as the test set. This procedure was repeated five times so that each sample obtained one out-of-fold (OOF) prediction. Meanwhile, to evaluate the performance of the DNN against simpler statistical models, a logistic regression model was constructed using the same set of biomarkers. A paired DeLong test was further applied to compare the ROC–AUC values between the DNN and logistic regression models on identical OOF samples.

### 2.6 Gene set enrichment analysis

To characterize the biological pathways associated with the identified biomarkers in AMI, gene set enrichment analysis (GSEA) was carried out using samples from the AMI and control groups in the GSE66360 training cohort. Spearman correlation coefficients between each biomarker and all other genes were computed with the R package “psych” (v 3.16.0) (26), and genes were ranked according to their correlation coefficients. Using the default background gene set “c2.cp.kegg_legacy.v2025.1.Hs.symbols” from “org.Hs.eg.db” (v 3.16.0) (20), enrichment analysis was performed with the R package “clusterProfiler” (v 4.2.2) (19), with thresholds set at |Normalized Enrichment Score (NES)| > 1 and adjusted *P* < 0.05. The top five enriched pathways with the lowest adjusted *P*-values for each biomarker were subsequently identified.

### 2.7 Immune infiltration analysis

Immune dysregulation is a key component of AMI pathophysiology, involving multiple immune cell populations such as macrophages, neutrophils, Tfh cells, CD8^+^ T cells, natural killer (NK) cells, Th1 cells, and tumor-infiltrating lymphocytes (TILs) (27). To quantify immune cell composition, the R package “GSVA” (v 1.46.0) (28) was applied to calculate enrichment scores for 28 immune cell types in AMI and control samples using the single-sample gene set enrichment analysis (ssGSEA) approach (29). Differences in immune cell abundance between the two groups were visualized with a heatmap. Immune cell subsets showing statistically significant differences between AMI and control groups were identified using Wilcoxon rank-sum tests (*P* < 0.05). In addition, Spearman correlation analysis was performed to examine relationships among differential immune cell types (|correlation coefficient (cor)| > 0.3, *P* < 0.05) with the R package “psych” (v 3.16.0) (26), and the resulting correlation patterns were displayed as a heatmap using pheatmap. To further explore immune–biomarker interactions, Spearman correlations between biomarker expression and significantly altered immune cell subsets were computed (*P* < 0.05, |cor| > 0.3), with results displayed in a lollipop chart to illustrate correlation strengths and directions.

### 2.8 Molecular regulatory network analysis

To investigate the role of transcription factors (TFs) in gene regulation, an mRNA–TF regulatory network was constructed to analyze the transcriptional regulatory effects of TFs on biomarkers, thereby revealing potential regulatory mechanisms. TFs related to biomarkers were predicted using the JASPAR database from the Network Analyst online site (https://www.networkanalyst.ca/NetworkAnalyst/uploads/ListUploadView.xhtml), and the complete TF–mRNA regulatory network was constructed using Cytoscape software (v 3.9.1) (30). In addition, the targeted microRNAs (miRNAs) for the biomarkers were predicted from the miRTarBase v9.0 and TarBase v9.0 databases using NetworkAnalyst, and the intersecting miRNAs were selected to obtain the final targeted miRNAs. Subsequently, Long non-coding RNAs (lncRNAs) potentially regulated by the selected miRNAs were retrieved from the miRNet platform (https://www.mirnet.ca/miRNet/home.xhtml), and for each miRNA, the five highest-ranked target lncRNAs were retained. These interactions were integrated to assemble a lncRNA–miRNA–mRNA regulatory network, which was visualized using Cytoscape (v 3.9.1) (30).

### 2.9 Drug prediction analysis and molecular docking

To identify candidate therapeutic compounds associated with the biomarkers, drug–gene interactions were queried using the R package “enrichR” (v 3.2) (31) based on the DSigDB database (http://tanlab.ucdenver.edu/DSigDB), with a significance cutoff of *P* < 0.05. To further validate the binding affinities of targeted drugs to biomarkers and enhance the reliability of the biomarker–drug interaction network, drugs demonstrating the highest combined score and the lowest *P*-value with the biomarkers were selected for subsequent molecular docking analyses. Three-dimensional structures of the selected compounds were obtained from the PubChem database (https://pubchem.ncbi.nlm.nih.gov/), while the corresponding protein structures encoded by the biomarkers were retrieved from the RCSB Protein Data Bank (https://www.rcsb.org/). These ligand and receptor structures were submitted to the cb-dock2 web server (https://cadd.labshare.cn/cb-dock2/php/index.php) to perform docking simulations and calculate binding scores. In this analysis, larger absolute docking scores indicate stronger predicted binding affinities and greater stability of the ligand–receptor complexes.

### 2.10 Reverse-transcription quantitative polymerase chain reaction (RT-qPCR)

Blood samples were collected from five patients with AMI and five healthy controls at the Second Hospital of Hebei Medical University. The study was conducted in accordance with the Declaration of Helsinki, received approval from the Second Hospital of Hebei Medical University (approval number: 2026-R728), and obtained written informed consent from all participants. Total RNA was extracted from the 10 samples using TRIzol reagent (Ambion, Austin, TX) and reverse-transcribed into cDNA using the SureScript First-Strand cDNA Synthesis Kit (Servicebio, Wuhan, China). RT-qPCR was performed with 2 × Universal Blue SYBR Green qPCR Master Mix (Servicebio, Wuhan, China); primer sequences are listed in Supplementary Table S1, with GAPDH as the endogenous reference gene. Gene expression levels were quantified using the 2^−*ΔΔ*Ct^ method (32). Quantitative PCR data were processed and visualized using GraphPad 10 software.

### 2.11 Statistical analysis

All computational analyses were performed using R software (version 4.3.1). Statistical comparisons between the AMI and control groups in the bioinformatics analyses were conducted using the Wilcoxon rank-sum test. For RT–qPCR results, statistical significance was determined using an unpaired *t*-test, with *P* < 0.05 considered statistically significant.

## 3 Results

### 3.1 Identification and functional analysis of 17 candidate genes

Differential expression analysis of the GSE66360 dataset identified 477 DEGs between the AMI group and controls (Figure 2A,B). Among these, 351 genes were upregulated, and 126 were downregulated in the AMI group (adjusted  *P*  <  0.05, |log_2_FC|  >  1) (Supplementary Table S2). The intersection of these DEGs with PTM-RGs identified 17 candidate genes (Figure 2C). GO enrichment analysis returned 425 significant terms (*P* <0.05), including 268 biological process (BP) terms such as polyubiquitination, response to virus, and negative regulation of response to external stimuli (Figure 2D, Supplementary Table S3). A total of 10 cellular component (CC) categories were significantly enriched, with prominent terms including the ubiquitin ligase complex, secretory granule lumen, and cytoplasmic vesicle lumen (Figure 2D, Supplementary Table S4). In addition, 43 molecular function (MF) terms were identified, mainly related to ubiquitin protein transferase activity, ubiquitin-like protein transferase activity, and ubiquitin protein ligase activity (Figure 2D, Supplementary Table S5) (Figure 2E, Supplementary Table S6). PPI network analysis highlighted 15 candidate genes with central roles in AMI. Protein NLRP3 exhibited interactions with PELI1 and TNFAIP3, suggesting that it may mediate key biological functions through these connections (Figure 2F). The identified candidate genes and pathways represent potential therapeutic targets and provide mechanistic insights into AMI.

**Figure 2:**
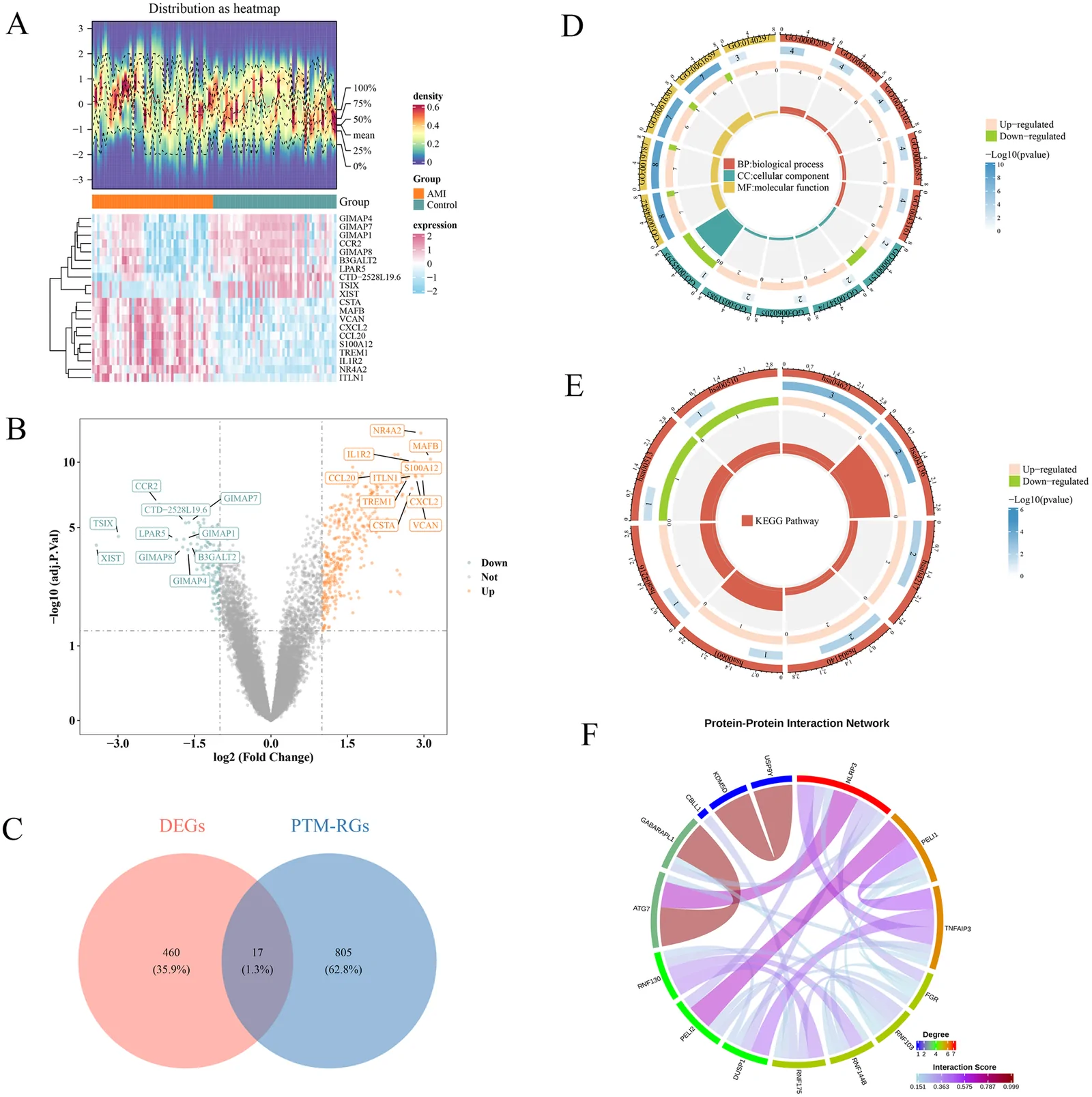
Identification and functional analysis of 17 candidate genes. **(A,B)** Differential expression analysis identified 477 DEGs between the AMI and control groups in the GSE66360 dataset. **(C)** The overlap between these DEGs and PTM RGs yielded 17 candidate genes. **(D)** GO enrichment analysis identified 425 significant biological process terms, 10 cellular component terms, and 43 molecular function terms associated with the candidate genes, with ubiquitin-related pathways being predominant. **(E)** Enrichment analysis identified 43 significant molecular function terms, predominantly related to ubiquitin-related enzymatic activities. **(F)** PPI network analysis identified 15 central candidate genes in AMI, with *NLRP3* forming high-confidence interactions with *PELI1* and *TNFAIP3*.

### 3.2 Acquisition of three biomarkers

Machine learning–based feature selection was applied to screen AMI-related biomarkers. Using the SVM-RFE approach with 10-fold cross-validation, an optimal subset of five genes was obtained, which achieved the lowest classification error and the highest prediction accuracy. This subset, defined as Feature Gene Set 1, comprised *GABARAPL1*, *PELI1*, *TNFAIP3*, *NLRP3*, and *B3GNT5* (Figure 3A). The XGBoost algorithm identified the top 12 most informative features, which comprised Feature Gene Set 2 (*TNFAIP3*, *GABARAPL1*, *PELI1*, *NLRP3*, *RNF175*, *CBLL1, KDM5D*, *B3GNT5*, *RNF103*, *ATG7*, *DUSP1*, and *USP9Y*; Figure 3B). RF analysis highlighted the five most important feature genes, defining Feature Gene Set 3 (*GABARAPL1*, *PELI1*, *TNFAIP3*, *NLRP3*, and *RNF175*) (Figure 3C,D). The intersection of the three feature sets revealed four overlapping candidate biomarkers: *GABARAPL1*, *PELI1*, *TNFAIP3*, and *NLRP3* (Figure 3E). Differential expression analysis in both the GSE66360 training cohort and the GSE61144 validation cohort showed that *GABARAPL1*, *PELI1*, and *NLRP3* were consistently upregulated in AMI samples relative to controls (*P* < 0.05), whereas *TNFAIP3* did not show stable differential expression across datasets. Accordingly, *GABARAPL1*, *PELI1*, and *NLRP3* were retained as the final set of biomarkers (Figure 3F,G). By integrating multiple machine learning strategies with diagnostic modeling, this framework offers a robust molecular basis for improving early detection in AMI. Finally, ROC analysis showed that the AUC values for the three biomarkers in both the GSE66360 training cohort and the GSE61144 validation cohort exceeded 0.80, indicating favorable diagnostic performance (Supplementary Figure 1A–F).

**Figure 3:**
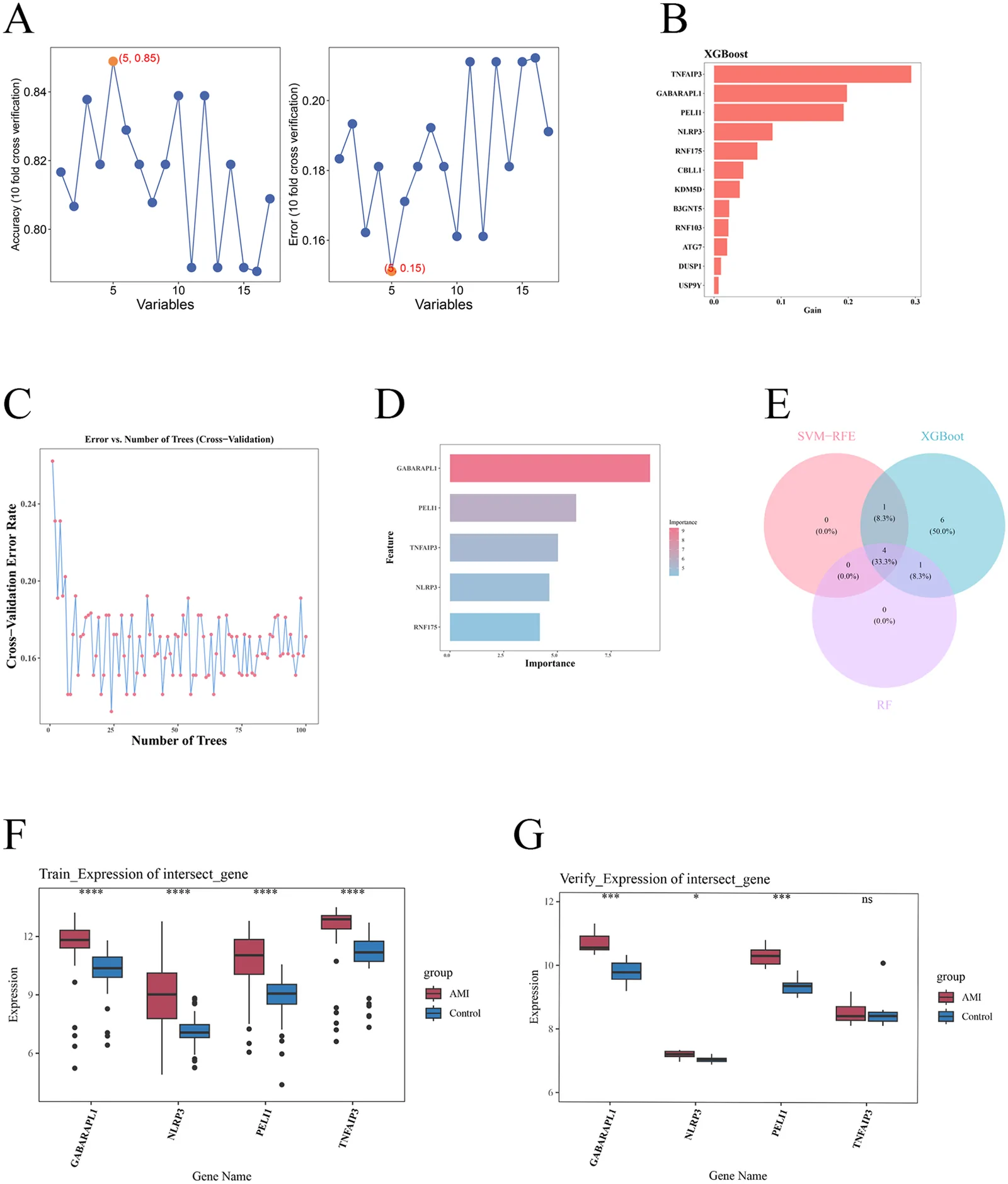
Acquisition of three biomarkers. **(A)** The SVM-RFE algorithm combined with 10-fold cross-validation identified an optimal 5-gene signature. **(B)** The XGBoost algorithm identified the top 12 most informative features. **(C,D)** RF analysis highlighted the five most important feature genes. **(E)** The intersection of the three feature sets revealed four overlapping candidate biomarkers. **(F,G)** Differential expression analysis was performed in both the GSE66360 training cohort and the GSE61144 validation cohort.

### 3.3 DNN model analysis and functional enrichment of biomarkers

The DNN model achieved an AUC of 0.963, indicating high discriminatory power between AMI and control samples (Figure 4A,B). Moreover, the cross-validation results showed that under fivefold cross-validation within GSE66360, the DNN model yielded an OOF AUC of 0.894 (95% confidence interval (CI): 0.832–0.957), whereas the logistic regression model achieved an AUC of 0.826 (95% CI: 0.736–0.916) (Figure 4C). The difference in AUC between the two models was not statistically significant (*P* = 0.1269).

**Figure 4:**
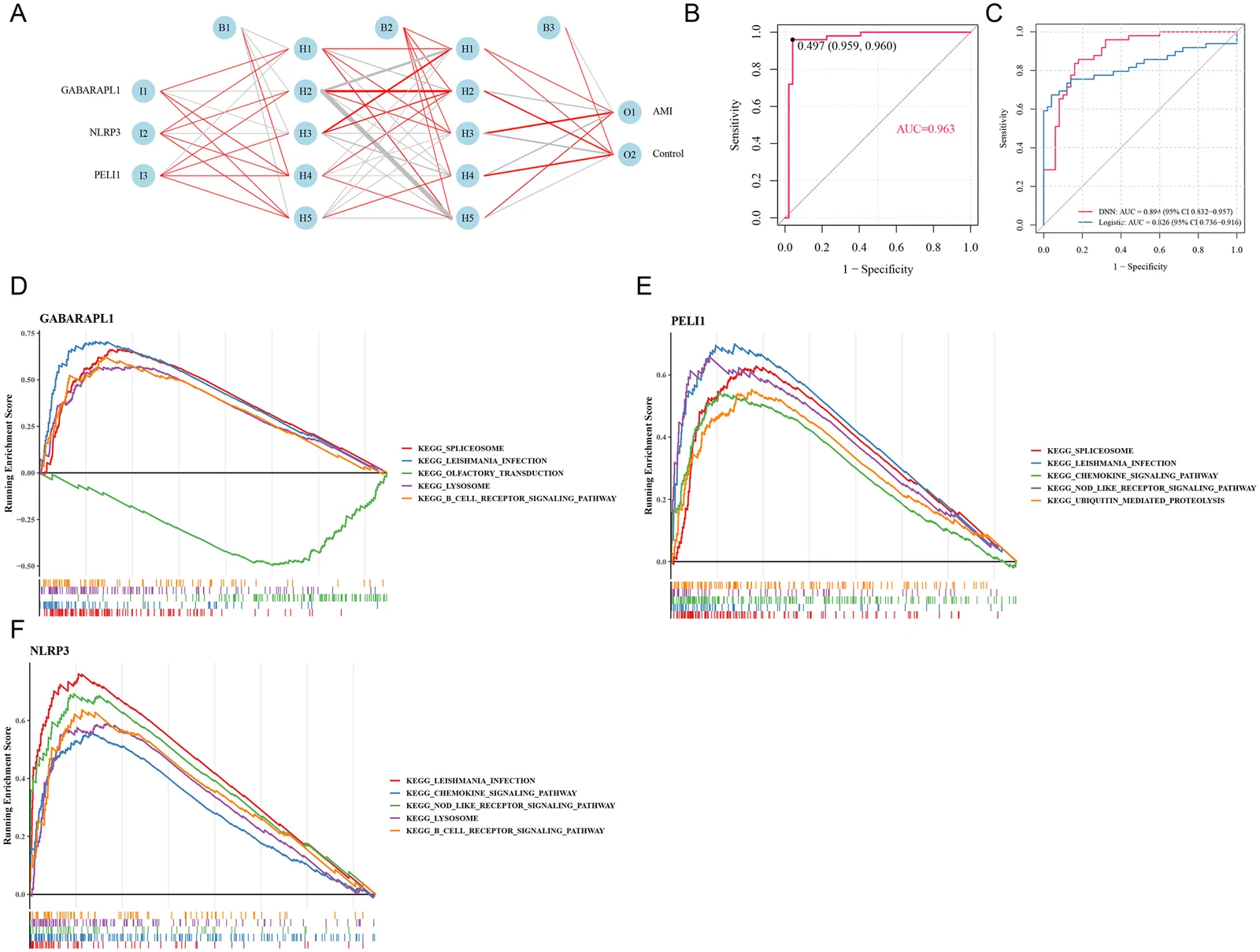
DNN model analysis and functional enrichment of biomarkers. **(A,B)** The DNN model achieved an AUC of 0.963 for discriminating AMI from control samples. **(C)** Performance comparison of DNN and logistic regression models via 5-fold stratified cross-validation in the GSE66360 cohort. **(D–F)** GSEA revealed significant enrichment of *GABARAPL1*, *PELI1*, and *NLRP3* in common pathways such as mRNA splicing and Leishmania infection (|NES| > 1, adj. *P* < 0.05).

To further elucidate the functional relevance of these biomarkers, GSEA was performed. *GABARAPL1* showed significant enrichment in 88 pathways related to mRNA splicing, Leishmania infection, and olfactory transduction (|NES| >1, adjusted *P* < 0.05) (Figure 4D, Supplementary Table S7). *PELI1* was enriched in 84 pathways including splicing, Leishmania infection, and chemokine signaling (Figure 4E, Supplementary Table S8). *NLRP3* was primarily associated with 76 pathways such as Leishmania infection, chemokine signaling, and non-canonical lymphocyte receptor signaling (Figure 4F, Supplementary Table S9). These findings provide functional insights into the selected biomarkers and support their potential role in the diagnosis of AMI.

### 3.4 Immune infiltration analysis

To delineate immune cell composition differences between AMI and control samples, central memory CD4⁺ T cells displayed the highest enrichment scores (Figure 5A). Statistical comparison using the Wilcoxon rank-sum test identified 23 immune cell subsets with significant differences in abundance between the two groups (*P* < 0.05), including activated B cells, activated CD4^+^ T cells, and activated dendritic cells (Figure 5B). Correlation analysis among differentially infiltrating immune cells showed a strong positive correlation between macrophages and neutrophils (cor =  0.87, *P* < 0.001) (Figure 5C, Supplementary Table S10). Correlation analysis between the biomarkers and immune cell subsets showed that *GABARAPL1* was positively associated with 18 immune cell types, with the strongest relationship observed for plasmablast-like dendritic cells (cor = 0.66, *P* < 0.001) (Figure 5D, Supplementary Table S10). Likewise, *PELI1* displayed positive correlations with 20 immune populations, again exhibiting the highest association with plasmablast-like dendritic cells (cor = 0.69, *P* < 0.001) (Figure 5E, Supplementary Table S11). *NLRP3* was positively linked to 19 immune cell subsets, with plasmablast-like dendritic cells showing the most prominent correlation (cor = 0.65, *P* < 0.05) (Figure 5F, Supplementary Table S11). Together, these findings underscore the involvement of *GABARAPL1*, *PELI1*, and *NLRP3* in immune-related processes and highlight their potential contributions to the immunopathological features of AMI, thereby providing a rationale for immune-targeted therapeutic strategies.

**Figure 5:**
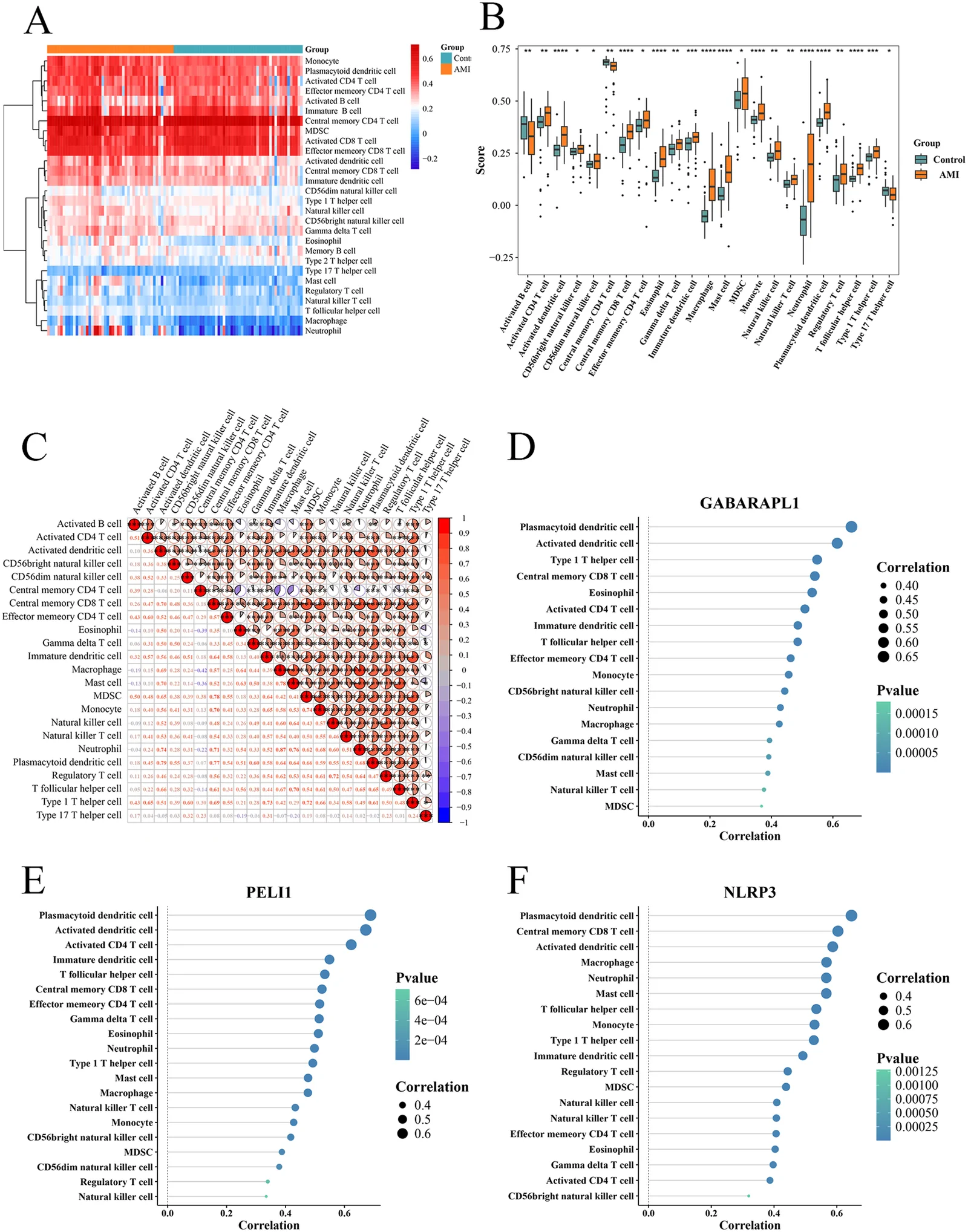
Immune infiltration analysis. **(A)** Analysis of immune cell composition revealed that central memory CD4^+^ T cells were the most enriched subset in AMI vs. control samples. **(B)** Significant abundance differences were detected in 23 immune cell subsets (e.g., activated B, CD4^+^ T, and dendritic cells; *P* < 0.05). **(C)** Correlation analysis among differentially infiltrated immune cells. **(D–F)** Correlation analyses revealed that *GABARAPL1*, *PELI1*, and *NLRP3* each exhibited the strongest correlation with plasmablast-like dendritic cells.

### 3.5 Regulatory network mapping and therapeutic target exploration of the identified biomarkers

TFs control gene expression by interacting with specific DNA motifs, thereby modulating mRNA production and governing key cellular processes such as proliferation, differentiation, and stress adaptation. To uncover potential regulatory mechanisms, we examined the transcriptional regulation of biomarkers by TFs. Our analysis predicted that *GABARAPL1*, *PELI1*, and *NLRP3* are regulated by 15, 7, and 14 TFs, respectively (Figure 6A). MicroRNAs (miRNAs) primarily modulate protein expression by binding to target mRNAs and participate in diverse developmental pathways and gene regulatory networks. Investigation of biomarker-associated miRNAs revealed that *NLRP3*, *GABARAPL1*, and *PELI1* are targeted by 1, 10, and 6 miRNAs, respectively, leading to the identification of 1,393 miRNA–lncRNA pairs in total (Figure 6B). Further analysis of biomarker interactions with potential AMI therapeutic drugs indicated that *GABARAPL1*, *PELI1*, and *NLRP3* are associated with 80, 29, and 42 potential drugs, respectively (*P* < 0.05) (Figure 6C, Supplementary Table S12). Subsequently, drugs with the highest combined score and the lowest P-value were selected for molecular docking with their corresponding biomarkers. The results showed docking scores of −7 kcal/mol for 1,4-chrysenequinone with *GABARAPL1* and −8.9 kcal/mol with *PELI1* (Figures 6D–G, Table 1). In addition, calcitriol exhibited a docking score of −10.1 kcal/mol when interacting with *NLRP3* (Figures 6H,I, Table 1), suggesting strong and stable ligand–receptor interactions. These computational results support the potential of these compounds as biomarker-targeted candidates and provide a rationale for subsequent experimental validation in cellular and animal models of AMI.

**Figure 6:**
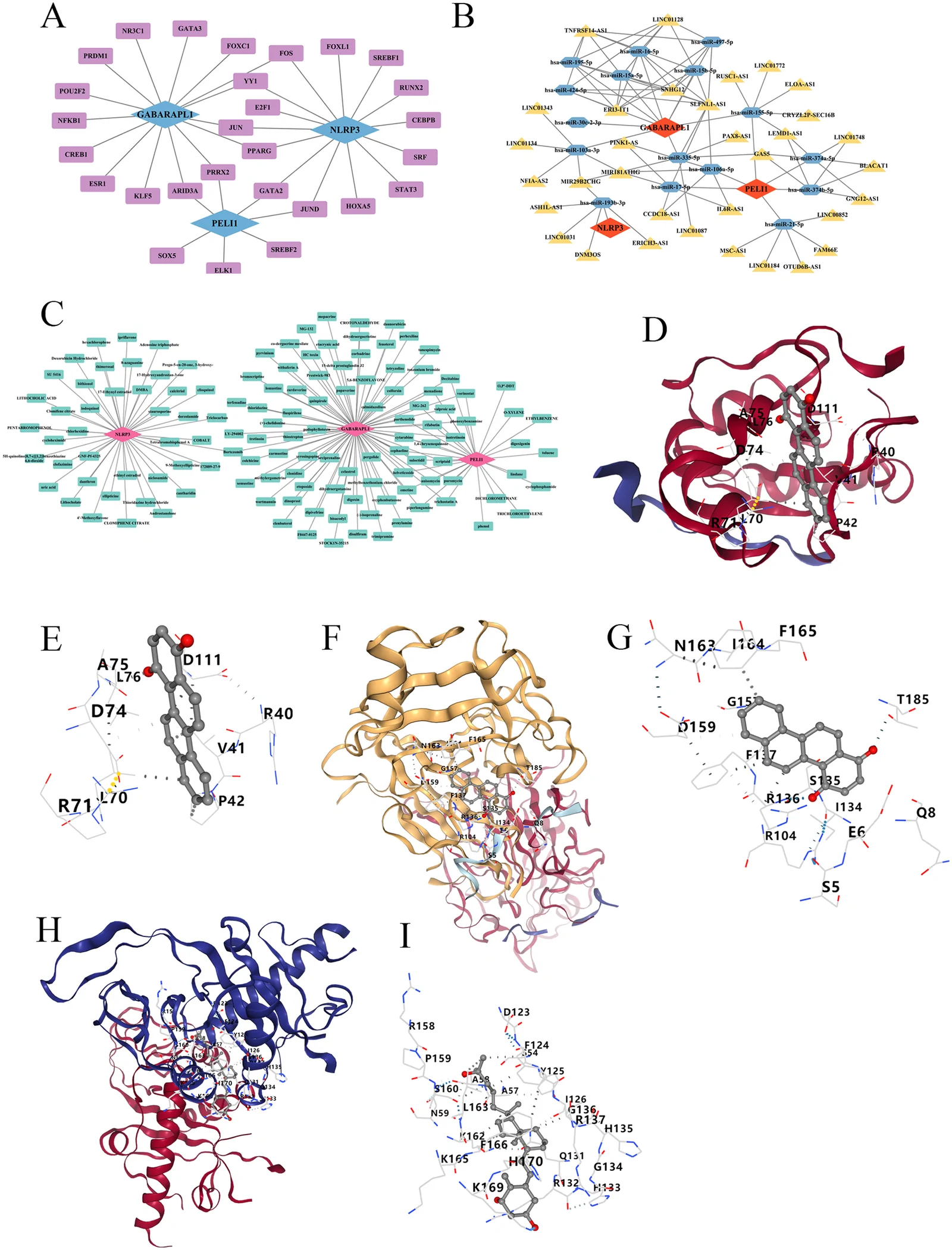
Biomarker regulatory network and therapeutic targets. **(A)** Transcriptional regulation analysis predicted that *GABARAPL1*, *PELI1*, and *NLRP3* are potentially regulated by 15, 7, and 14 TFs, respectively. **(B)** miRNA biomarker analysis revealed that *NLRP3*, *GABARAPL1*, and *PELI1* are targeted by 1, 10, and 6 miRNAs, respectively, leading to the identification of 1,393 miRNA–lncRNA pairs. **(C)** Further analysis of biomarker interactions with potential AMI therapeutic drugs. **(D–G)** Molecular docking revealed strong binding of 1,4-chrysenequinone to *PELI1* and *GABARAPL1*. **(H,I)** Calcitriol docked to NLRP3 with a score of −10.1 kcal/mol, indicating predicted strong binding affinity in the simulation.

**Table 1:** Molecular docking scores of prioritized compounds with target biomarkers.

Drug	PubChem CID	Biomarker	PDB ID	Vina score (Kcal/mol)
1,4-chrysenequinone	180,933	*GABARAPL1*	8 × 8a	−7
1,4-chrysenequinone	180,933	*PELI1*	9if9	−8.9
calcitriol	5,280,453	*NLRP3*	4b5p	−10.1

### 3.6 Validation of the expression of key genes

Validation of the transcriptomic findings was performed using RT–qPCR to quantify the expression levels of *NLRP3*, *PELI1*, and *GABARAPL1* in clinical blood samples from the AMI and control groups (Figure 7). The results confirmed that all three genes were significantly upregulated in the AMI group compared with controls (*P* < 0.001).

**Figure 7:**
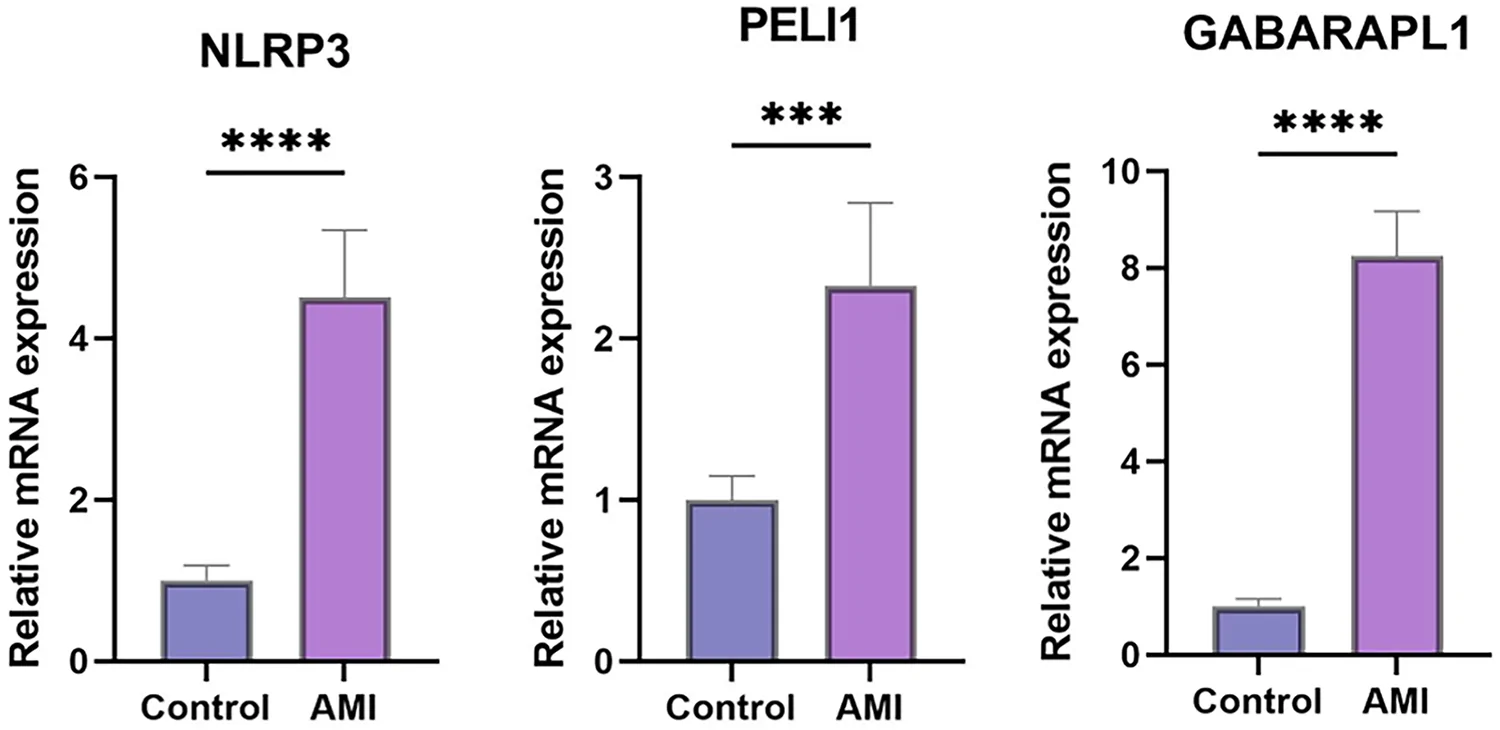
RT-qPCR validation of key genes. *** indicates that *P* < 0.001, **** represents *P* < 0.0001.

## 4 Discussion

AMI is a life-threatening condition caused primarily by acute coronary occlusion following atherosclerotic plaque rupture, leading to severe myocardial ischemia and necrosis (33, 34). While traditionally more prevalent in elderly males, its incidence is rising among younger individuals (35). Despite therapeutic advances that have improved survival, the pathophysiology and long-term prognosis of AMI remain incompletely understood. Emerging evidence underscores the importance of PTMs in regulating cellular activities and intercellular communication, processes implicated in various diseases (36–38). Nevertheless, the specific links and mechanistic roles of PTMs in AMI remain incompletely defined. Based on transcriptomic data, our study aimed to investigate biomarkers derived from PTM-regulatory genes and the regulatory mechanisms in AMI. Through a series of bioinformatics analyses, three key biomarkers were identified: *GABARAPL1*, *PELI1*, and *NLRP3*, and a comprehensive mechanistic exploration was subsequently conducted to elucidate the functional roles of the identified biomarkers. Together, these results highlight the importance of PTM-related mechanisms in AMI and identify relevant molecular targets for improving early diagnosis.

GABA type A receptor-associated protein like 1 (*GABARAPL1*), a member of the autophagy-related protein family, plays a crucial role in autophagosome formation and maturation (39). Previous reports indicate that *GABARAPL1* is differentially expressed in AMI and participates in ferroptosis-related mechanisms (40). By interacting with LC3 family proteins, *GABARAPL1* helps coordinate autophagy, linking cellular stress responses to damage repair processes relevant to AMI (41). In our analysis, *GABARAPL1* was significantly upregulated in AMI samples, suggesting an activated autophagic response that may help maintain cellular homeostasis by clearing damaged organelles (41). Notably, the functionality of *GABARAPL1* is critically dependent on PTMs. Specifically, it forms a covalent thioester bond with the E2-conjugating enzyme ATG3 via its C-terminus. Recent studies have further revealed a novel non-covalent “backside” interaction between the two molecules, which is essential for ensuring the accuracy and efficiency of autophagic modification (42). Therefore, *GABARAPL1* may serve as a key molecular nexus linking the PTM regulatory network to cardioprotective mechanisms in AMI, providing a new direction for exploring the pathological mechanisms and potential therapeutic targets in AMI.

*PELI1*, encoding a member of the Pellino family of E3 ubiquitin ligases, participates in ubiquitin-mediated regulation of multiple signaling cascades, including the NF–κB and JAK/STAT pathways, and plays an important role in shaping inflammatory and immune signaling (43, 44). In our analysis, *PELI1* expression was markedly elevated in peripheral blood samples from patients with AMI, implicating this molecule in the disease-associated regulatory network. Existing research indicates that *PELI1* plays multiple regulatory roles in cardiovascular diseases. During myocardial ischemia/reperfusion injury, macrophage-specific knockout of *PELI1* alleviates myocardial damage, a mechanism linked to inhibition of M1 macrophage polarization (45). Furthermore, deficiency of *PELI1* has been shown to delay the progression of atherosclerosis, further supporting its critical role in cardiovascular inflammation and injury (46). Collectively, this evidence suggests that *PELI1* may contribute to the onset and progression of AMI by regulating inflammatory responses, autophagy, and immune cell polarization. In addition, as a key enzyme regulating protein ubiquitination, *PELI1* mediates the ubiquitination of specific substrates—such as influencing cell fate by regulating the polyubiquitination of BCL6 (47)—and is critically involved in inflammatory and immune-related processes. Therefore, *PELI1* may drive the inflammatory and cellular damage processes in AMI through ubiquitination, a critical form of PTM.

*NLRP3*, a key member of the NLR family, forms an essential component of the inflammasome machinery and is strongly implicated in inflammatory activation during AMI. Activation of *NLRP3* triggers caspase-1–dependent signaling, leading to the maturation and secretion of IL-1β and IL-18 and thereby amplifying inflammatory cascades that drive cardiomyocyte apoptotic and necroptotic cell death (48, 49). Accumulating evidence strongly associates *NLRP3* activation with myocardial injury, highlighting its role in local inflammation during AMI progression. Various PTMs of *NLRP3*, including ubiquitination, deacetylation, and phosphorylation, have been linked to AMI pathology (50, 51). Consistent with this, our results showed significantly upregulated *NLRP3* expression in patients with AMI, supporting its role in promoting inflammatory myocardial damage. Targeted inhibition of *NLRP3* may therefore represent a promising therapeutic strategy for AMI.

Our findings suggest a potential regulatory network connecting *GABARAPL1*, *PELI1*, and *NLRP3*. *PELI1* has been reported to modulate *NLRP3* inflammasome activity by controlling its ubiquitination status, thereby influencing downstream inflammatory signaling (52, 53). Moreover, *GABARAPL1*-mediated autophagy may modulate *PELI1* and *NLRP3* protein stability and activity, indicating a complex interplay among autophagy, ubiquitination, and inflammasome activation. Unraveling these connections is essential for clarifying the underlying molecular pathophysiology of AMI.

Pathway enrichment analysis indicated that all three biomarkers were associated with multiple immune-related signaling pathways, including those involved in *Leishmania* infection and chemokine-mediated communication. During the pathological progression of AMI, chemokine signaling is strongly activated, mediating the recruitment of inflammatory cells such as neutrophils and monocytes/macrophages to the ischemic myocardium. This directly exacerbates local inflammation, cardiomyocyte apoptosis, and tissue remodeling (54, 55). For instance, targeting CXCR4 can promote regulatory T-cell mobilization, aiding postmyocardial infarction tissue repair (56), while dysregulation of the IL-6/MCP-1/STAT3 axis represents a potential strategy for intervening in postinfarction inflammation (57), highlighting the importance of this pathway as a therapeutic target. Notably, the activity and specificity of the chemokine signaling pathway are precisely regulated by various PTMs (58). For example, the activity of the chemokine receptor CXCR2 can be modulated through ubiquitination at K327 (59), and chemokine genes are also subject to epigenetic modifications (60). This suggests that the key biomarkers identified in this study—*PELI1* (an E3 ubiquitin ligase) and *NLRP3* (a core inflammasome component)—may be involved in regulating immune cell recruitment and inflammatory cascades in AMI by influencing PTMs, such as ubiquitination, of chemokine-related proteins. Accordingly, examining chemokine signaling through a PTM-focused framework offers new insights into the immunopathological processes of AMI.

Correlation profiling between the identified biomarkers and immune cell subsets showed that *GABARAPL1*, *PELI1*, and *NLRP3* were most strongly associated with plasmacytoid dendritic cells (pDCs) (cor > 0.30, *P* < 0.001). pDCs represent a distinct dendritic cell population characterized by their capacity to rapidly secrete type I interferons (IFN-α/β) and to coordinate innate and adaptive immune activation, particularly during antiviral and inflammatory responses (61, 62). The functional state of pDCs influences various disease processes, including autoimmunity, inflammation, and cancer. In the context of cardiovascular diseases, the role of pDCs is complex. On the one hand, studies indicate that pDCs can exert protective effects against atherosclerosis by modulating T-cell proliferation and activity (63). On the other hand, dendritic cells, as key antigen-presenting cells, contribute significantly to cardiovascular inflammatory processes such as atherosclerotic plaque formation and destabilization through their activation and migration (64). The strong association found in this study between PTM-related biomarkers and pDCs suggests that PTMs may act as a critical switch regulating pDC functionality in AMI. For instance, the transcriptional activity of IRF7, a master transcription factor for Type I interferon production in pDCs, is negatively regulated by SUMOylation (65). Therefore, *GABARAPL1* (involved in autophagy), *PELI1* (an E3 ubiquitin ligase), and *NLRP3* (inflammasome) may modulate pDC-mediated inflammation in AMI through PTM-dependent mechanisms. However, whether these biomarkers directly regulate pDC function via specific PTMs in AMI remains to be experimentally validated.

In summary, our study systematically identified *GABARAPL1*, *PELI1*, and *NLRP3* as potential AMI biomarkers using a range of bioinformatics approaches. The developed predictive model provides a foundation for early diagnosis and therapeutic exploration. However, several limitations should be acknowledged. First, the bioinformatics analyses were based on publicly available transcriptomic datasets with a modest sample size, which may not fully represent the heterogeneity of AMI across different ethnic backgrounds, geographic regions, or comorbid conditions, potentially limiting the broader applicability of the results. In addition, this study only performed gene expression validation based on transcriptomic profiling and RT-qPCR. Notably, the RT–qPCR validation included only five patients with AMI and five healthy controls. This limited sample size precludes drawing robust conclusions about population-level expression differences or clinical diagnostic utility of biomarkers. We are also lacking direct protein-level measurements (such as ELISA or Western blot). Future work using proteomic approaches or targeted protein assays, together with larger-scale independent clinical cohorts, will further verify the protein expression changes of these biomarkers and their association with the pathophysiological processes of AMI. Moreover, although potential drugs have been predicted and subjected to molecular docking, their pharmacological activity, specificity, and safety require further experimental confirmation. Future studies should establish relevant cellular and animal models to rigorously examine the functional roles and mechanisms of these biomarkers in AMI. Third, our analysis relies entirely on transcriptomic data and a pre-compiled PTM-regulator gene set. We did not perform proteomic or PTM-enrichment experimental assays to directly measure protein post-translational modification events in AMI samples. Alterations in mRNA levels of PTM regulatory genes do not necessarily correspond to changes in protein abundance or actual PTM modification status of target substrates. Therefore, our findings represent bioinformatic predictions at the transcriptional level. Further proteomic and PTM-omic experiments in clinical AMI samples and preclinical animal models are required to verify whether these biomarkers indeed drive PTM alteration during AMI progression. Further exploration of the complex interactions among these biomarkers within regulatory networks will yield a more comprehensive understanding of AMI pathogenesis and support the development of effective multi-target therapeutic strategies.

## Data Availability

The datasets presented in this study can be found in online repositories. The names of the repository/repositories and accession number(s) can be found in the article/Supplementary Material.

## Supplementary material

Supplementary Figure 1 **Characteristic (ROC) analysis of biomarkers in GSE66360 and GSE61144 datasets.** (A-C) The ROC results of *GABARAPL1*, *PELI1*, and *NLRP3* in GSE66360 dataset. (D-F) The ROC results of *GABARAPL1*, *PELI1*, and *NLRP3* in GSE61144 dataset.
